# Supplementary figures and images for: Fusion of histone variants to Cas9 suppresses non-homologous end joining
Source: PLoS One. 2024 May 13;19(5):e0288578. doi: 10.1371/journal.pone.0288578 (PMC11090291; doi:10.1371/journal.pone.0288578)

S2 Fig.

A

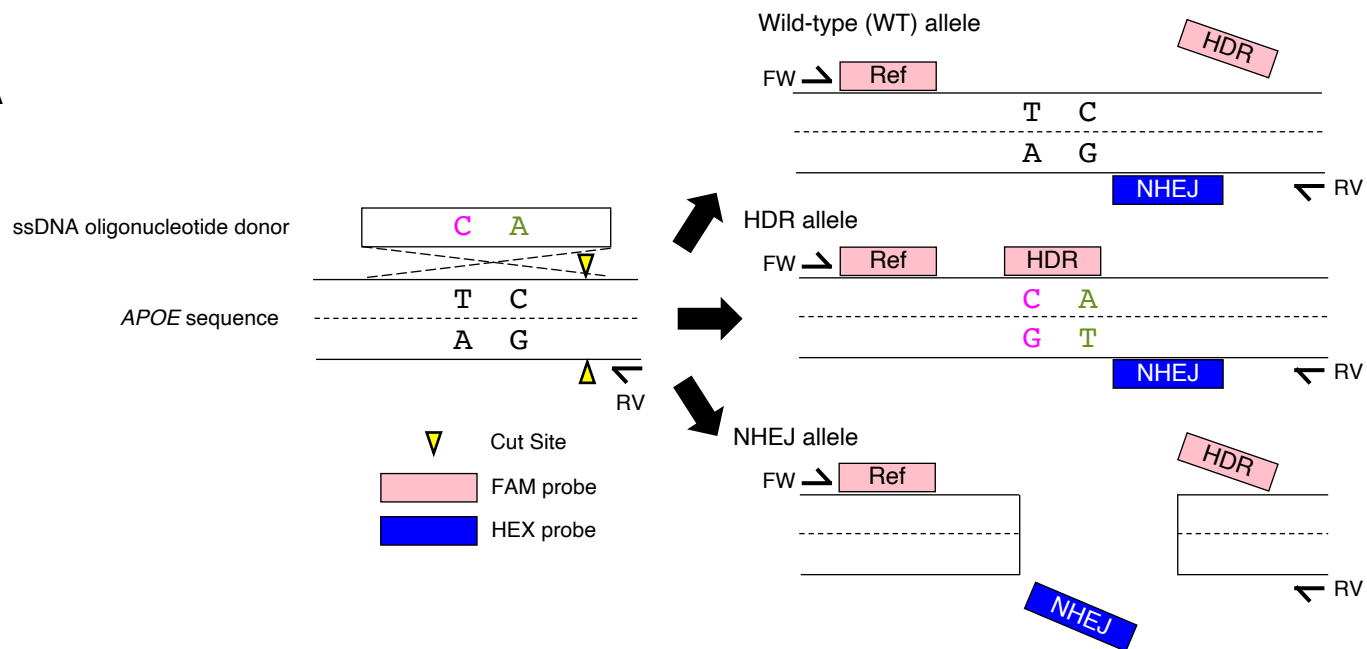

B

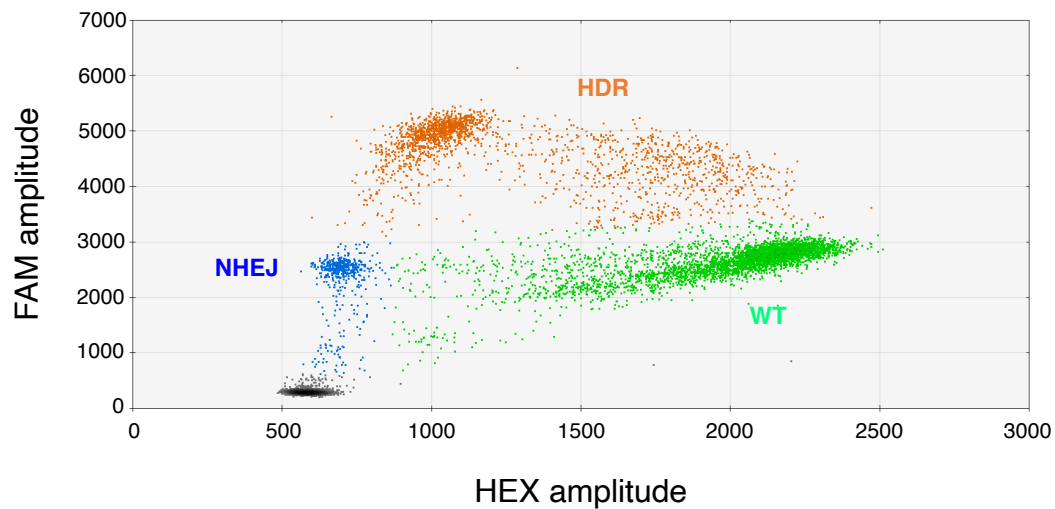

Supplement: S2 Fig — (PDF) [file pone.0288578.s002.pdf]

S3 Fig.

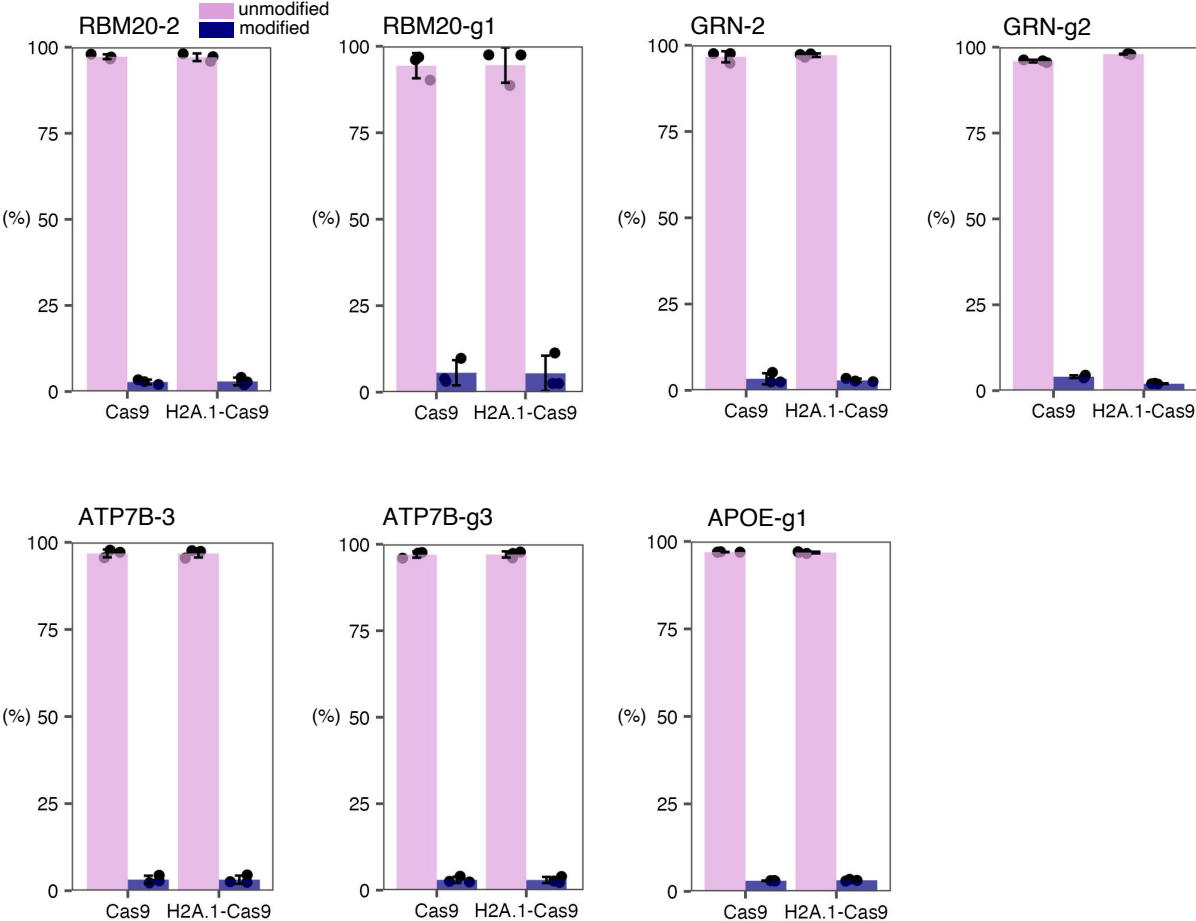

Supplement: S3 Fig — (PDF) [file pone.0288578.s003.pdf]
