# Supplementary material for: Fusion of histone variants to Cas9 suppresses non-homologous end joining
Source: PLoS One. 2024 May 13;19(5):e0288578. doi: 10.1371/journal.pone.0288578 (PMC11090291; doi:10.1371/journal.pone.0288578)
Supplement: S1 Table — (PDF) [file pone.0288578.s004.pdf]

S1 Table. Oligonucleotides used for plasmid construction in this study.

Name	Sequence (5'-3')	Plasmid
BamHICassette Fw	gATCCACGGAGTCCCtGCAGC	N-GS
BamHICassette Rv	CGACCTTCCGCTTCTTCTTT	
N-GS-Cas9 Fw	GGTGGCGGTGGATCCGACAAGAAGTACAGCATCGG	
BamHICassette Rv2	GGCTGCaGGGACTCCGTGGA	
BamHI-H2A.X Fw	GgATCCACGGAGTCCCtGCAGCCTCGGGCCGCGGCAAGACTGG	
BamHI-GGGGS-H2A.X Rv	GGATCCACCGCCACCGTACTCCTGGGAGGCCTGGG	
N-GS3-Cas9 Fw	GGTGGCGGTGGAAGTGGTGGCGGTGGATCTGACAAGAAGTACAGCATCGG	N-GS3
N-GS3-H2A.X Rv	GGATCCACCGCCACCGTACT	
N-GS5-Cas9 Fw	GGCGGTGGAGGCAGTGGAGGCGGTGGATCTGACAAGAAGTACAGCATCGG	N-GS5
N-GS5 Rv	AGATCCACCGCCACCACTTC	
FseI-GS-H2A.X Fw	GGCCGGCCAGGCAAAAAAGAAAAAGGGTGGCGGTGGATCCTCGGGCCGCGGCAAGACTGG	C-GS
FseI-GS3-H2A.X Fw	GGCCGGCCAGGCAAAAAAGAAAAAGGGCGGTGGAGGCTCCGGCGGAGGAGGTAGTGGAGGTGGAGGATCTTCGGGCCGCGGCAAGACTGG	C-GS3
FseI-GS5-H2A.X Fw	GGCCGGCCAGGCAAAAAAGAAAAAGGGCGGTGGAGGCTCCGGCGGAGGAGGTAGTGGAGGTGGAGGATCTTCGGGCCGCGGCAAGACTGG	C-GS5
EcoRI-H2A.XRv	GAATTCGTACTCCTGGGAGGCCTGGG	C-GS/C-GS3/ C-GS5
AgeI-kozak-BsdR Fw	ACCGGTGCCACCATGAAAACCTTCAACATCTC	pGB
EcoRI-BsdR Rv	GAATTCTTAATTTCGGGTATATTTGA	
H2A.X_K5Q Fw	cAGACTGGCGGCAAGGCCCGC	KQ-Cas9
H2A.X_K5R Rv	gGACTGGCGGCAAGGCCCGCG	KR-Cas9
H2A.X_K5Q/K5R Rv	GCCGCGGCCCGAGGCTGCAG	KQ-Cas9/KR-Cas9
H2A.X_S139D Fw	gaCCAGGAGTACGGTGGCGGTG	SD-Cas9
H2A.X_S139A Fw	gCCCAGGAGTACGGTGGCGGT	SA-Cas9
H2A.X_S139A/S139D Rv	GGCCTGGGTGGCCTTCTTGC	SD-Cas9/SA-Cas9
H2A.X_K134L Fw	ttGAAGGCCACCCAGGCCTCCC	KL-Cas9
H2A.X_K134M Fw	tGAAGGCCACCCAGGCCTCCC	KM-Cas9
H2A.X_K134A Fw	gcGAAGGCCACCCAGGCCTCCC	KA-Cas9
H2A.X_K134L/K134A/K134LS139D/K134AS139D Rv	GCCGCCCGAGGGCGCCTTCG	KL-Cas9/KA-Cas9/KL_SD-Cas9/KA_SD-Cas9
H2A.X_K134M Rv	TGCCGCCCGAGGGCGCCTTC	KM-Cas9
H2A.X_K134LS139D Fw	ttGAAGGCCACCCAGGCCgaCC	KL_SD-Cas9
H2A.X_K134MS139D Fw	tGAAGGCCACCCAGGCCgaCC	KM_SD-Cas9
H2A.X_K134AS139D Fw	gcGAAGGCCACCCAGGCCgaCC	KA_SD-Cas9
*BamHI-H2A.1-GS3-BamHI	AGAAGCGGAAGGTCGGgATCCACGGAGTCCCTGCAGCC**TCGGGACGCGGCAAGCAGGGAGGCAAAGCTCGCGCCAAAGCCAAGACCCGCTCTTCTCGTGCCGGTCTCCAGTTCCCCGTGGGCCGAGTGCACCGACTGCTCCGCAAGGGCAACTATGCTGAGCGGGTCGGGGCCGGCGCGCCGGTGTACCTGGCGGCGGTGCTGGAGTACCTGACTGCCGAGATCCTGGAGCTGGCGGGCAACGCCGCCCGCGACAACAAGAAGACCCGCATTATCCCGCGCCACTTGCAGCTGGCCATCCGCAACGACGAGGAGCTCAACAAGCTGCTGGGCAAAGTAACCATCGCTCAGGGTGGTGTCCTGCCCAACATCCAGGCTGTGCTACTGCCCAAGAAGACCGAGAGTCACCACAAGGCCAAAGGCAAA**GGTGGCGGTGGATCCGACAAGAAGTACAGC	H2A.1-Cas9
*BamHI-H2A.Z-GS3-BamHI	AGAAGCGGAAGGTCGGgATCCACGGAGTCCCTGCAGCC**GCTGGCGGTAAGGCTGGAAAGGACTCCGGAAAGGCCAAGACAAAGGCGGTTTCCCGCTCGCAGAGAGCCGGCTTGCAGTTCCCAGTGGGCCGTATTCATCGACACCTAAAATCTAGGACGACCAGTCATGGACGTGTGGGCGCGACTGCCGCTGTGTACAGCGCAGCCATCCTGGAGTACCTCACCGCAGAGGTACTTGAACTGGCAGGAAATGCATCAAAAGACTTAAAGGTAAAGCGTATTACCCCTCGTCACTTGCAACTTGCTATTCGTGGAGATGAAGAATTGGATTCTCTCATCAAGGCTACAATTGCTGGTGGTGGTGTCATTCCACACATCCACAAATCTCTGATTGGGAAGAAAGGACAACAGAAGACAGTC**GGTGGCGGTGGATCCGACAAGAAGTACAGC	H2A.Z-Cas9
*BamHI-macroH2A.1-GS3-BamHI	AGAAGCGGAAGGTCGGgATCCACGGAGTCCCTGCAGCC**TCGAGCCGCGGTGGGAAGAAGAAGTCCACCAAGACCTCCAGGTCTGCCAAAGCAGGAGTCATCTTTCCCGTGGGGCGGATGCTGCGGTACATCAAGAAAGGCCACCCCAAGTACAGGATTGGAGTGGGGGCACCCGTGTACATGGCCGCCGTCCTGGAATACCTGACAGCGGAGATTCTGGAGCTGGCTGGCAATGCAGCGAGAGACAACAAGAAGGGACGGGTCACACCCCGGCACATCCTGCTGGCTGTGGCCAATGATGAAGAGCTGAATCAGCTGCTAAAAGGAGTCACCATAGCCAGTGGGGGTGTGTTACCCAACATCCACCCCGAGTTGCTAGCGAAGAAGCGGGGATCCAAAGGAAAGTTGGAAGCCATCATCACACCACCCCCAGCCAAAAAGGCCAAGTCTCCATCCCAGAAGAAGCCTGTATCTAAAAAAGCAGGAGGCAAGAAAGGGGCCCGGAAATCCAAGAAGAAGCAGGGTGAAGTCAGTAAGGCAGCCAGCGCCGACAGCACAACCGAGGGCACACCTGCCGACGGCTTCACAGTCCTCTCCACCAAGAGCCTCTTCCTTGGCCAGAAGCTGAACCTTATTCACAGTGAAATCAGTAATTTAGCCGGCTTTGAGGTGGAGGCCATAATCAATCCTACCAATGCTGACATTGACCCTAAAGATGACCTAGGAAACACGCTGGAGAAGAAAGGTGGCAAGGAGTTTGTGGAAGCTGTCTTGGAACTCCGGAAAAAGAACGGGCCCTTGGAAGTAGCTGGAGCTGCTGTCAGCGCAGGCCATGGCCTGCCTGCCAAGTTTGTGATCCACTGTAATAGTCCAGTTTGGGGTGCAGACAAGTGTGAAGAACTTCTGGAAAAGACAGTGAAAAACTGCTTGGCCCTGGCTGATGATAAGAAGCTGAAATCCATTGCATTTCCATCCATCGGCAGCGGCAGGAACGGTTTTCCAAAGCAGACAGCAGCTCAGCTGATTCTGAAGGCCATCTCCAGTTACTTCGTGTCTACAATGTCCTCTTCCATCAAAACGGTGTACTTCGTGCTTTTTGACAGCGAGAGTATAGGCATCTATGTGCAGGAAATGGCCAAGCTGGACGCCAAC**GGTGGCGGTGGATCCGACAAGAAGTACAGC	macroH2A.1-Cas9

*BamHI-H2A.B-GS3-BamHI	AGAAGCGGAAGGTCGGgATCCACGGAGTCCCTGCAGCC**CCGAGGAGGAGGAGACGCCGAGGGTCCTCCGGTGCTGGCGGCCGGGGGCGGACCTGCTCTCGCACCGTCCGAGCGGAGCTTTCGTTTTCAGTGAGCCAGGTGGAGCGCAGTCTACGGGAGGGCCACTACGCTCAGCGCCTGAGTCGCACGGCGCCGGTCTACCTCGCTGCGGTTATTGAGTACCTGACGGCCAAGGTCCCGGAGCTGGCGGGCAACGAGGCCCAGAACAGCGGAGAGCGGAACATCACTCCCCTGCTGCTGGACATGGTGGTTCACAACGACAGGCTACTGAGCACCCTTTTCAACACGACCACCATCTCTCAAGTGGCCCCTGGCGAGGAC**GGTGGCGGTGGATCCGACAAGAAGTACAGC	H2A.B-Cas9
*BamHI-H2B-GS3-BamHI	AGAAGCGGAAGGTCGGgATCCACGGAGTCCCTGCAGCC**CCGGAcCCAGCGAAATCCGCTCCTGCTCCCAAGAAGGGCTCCAAAAAGGCTGTTACGAAAGTGCAGAAGAAGGACGGCAAGAAGCGCAAGCGCAGCCGCAAGGAGAGCTACTCCGTTTACGTGTACAAGGTGCTGAAGCAGGTCCACCCCGACACCGGCATCTCGTCCAAGGCCATGGGCATCATGAACTCCTTCGTCAACGACATCTTCGAGCGCATCGCGGGAGAGGCGTCCCGCCTGGCGCACTACAACAAGCGCTCCACCATCACATCCCGCGAGATCCAGACGGCCGTGCGCCTGCTGCTGCCCGGCGAGCTGGCCAAGCACGCCGTGTCCGAGGGCACCAAGGCGGTCACCAAGTACACCAGCTCGAACCCGAGGAATCTGTCTCCAACTAAGCCTGGTGGCAGTGAGGACCGTCAGCCCCCTCCCAGCCAGCTGTCAGCTATACCTCCATTCTGTCTGGTTCTCAGGGCTGGAATCGCTGGGCAGGTC**GGTGGCGGTGGATCCGACAAGAAGTACAGC	H2B-Cas9
*BamHI-H3.3-GS3-BamHI	AGAAGCGGAAGGTCGGgATCCACGGAGTCCCTGCAGCC**GCCCGAACCAAGCAGACTGCTCGTAAGTCCACAGGTGGGAAAGCCCCCCGCAAACAGCTGGCCACGAAAGCCGCCAGGAAAAGCGCTCCCTCTACCGGCGGGGTGAAGAAGCCTCATCGCTACAGGCCCGGGACCGTGGCGCTTCGAGAGATTCGTCGTTATCAGAAGTCGACCGAGCTGCTCATCCGGAAGCTGCCCTTCCAGAGGTTGGTGAGGGAGATCGCGCAGGATTTCAAAACCGACCTGAGGTTTCAGAGCGCAGCCATCGGTGCGCTGCAGGAGGCTAGCGAAGCGTACCTGGTGGGTCTGTTCGAAGATACCAACCTGTGTGCCATCCACGCTAAGAGAGTCACCATCATGCCCAAAGACATCCAGTTGGCTCGCCGGATACGGGGAGAGAGAGCT**GGTGGCGGTGGATCCGACAAGAAGTACAGC	H3.3-Cas9
H31R Fw	gCCGACTGCTCCGCAAGGGCA	H2A-Cas9
H31R Rv	GCACTCGGCCCACGGGGAAC	
T16S Fw	gCCGCTCTTCTCGTGCCGGTC	H2A.2-Cas9/H2A.L-Cas9/H2A.J-Cas9
T16S Rv	TCTTGGCTTTGGCGCGAGCT	
L51M Fw	aTGGCGGCGGTGCTGGAGTAC	H2A.2-Cas9
L51M Rv	GTACACCGGCGCGCCGGCCC	
K99R Fw	gAGTAACCATCGCTCAGGGTG	H2A.L-Cas9
K99R Rv	TGCCCAGCAGCTTGTTGAGC	
A10V Fw	tTCGCGCCAAAGCCAAGACCC	H2A.J-Cas9
A10V Rv	CTTTGCCTCCCTGCTTGCCG	
c-terminus Fw	cagaagacgaagagcaaaGGTGGCGGTGGATCCGGTGG	
c-terminus Rv	ACTCTCGGTCTTCTTGGGCA	
S31A Fw	gCTACCGGCGGGGTGAAGAAG	H3.2-Cas9
S31A Rv	GGGAGCGCTTTTCCTGGCGG	
A87S_I89V_G90M Fw	tCAGCCgTCatgGCGCTGCAGGAGGCTAGCGA	
A87S_I89V_G90M Rv	GCTCTGAAACCTCAGGTCGG	
S96C Fw	tGCGAAGCGTACCTGGTGGGT	H3.1-Cas9
S96C Rv	AGCCTCCTGCAGCGCCATGA	
A24V Fw	tCGCCAGGAAAAGCGCTCCCG	H3-Cas9
A24V Rv	CTTTCGTGGCCAGCTGTTTG	
V71M Fw	aTGAGGGAGATCGCGCAGGAT	
V71M Rv	CAACCTCTGGAAGGGCAGCT	

Recognition sequences of restriction endonuclease are underlined.

Lower case letters show intended mutations.

Bold letters indicate gene-specific oligonucleotides.

Asterisks indicate that the oligonucleotides were synthesized by gBlocks Gene Fragments from IDT.
