# Supplementary material for: Fusion of histone variants to Cas9 suppresses non-homologous end joining
Source: PLoS One. 2024 May 13;19(5):e0288578. doi: 10.1371/journal.pone.0288578 (PMC11090291; doi:10.1371/journal.pone.0288578)
Supplement: S2 Table — (PDF) [file pone.0288578.s005.pdf]

S2 Table. Target genes and mutations engineered in this study.

Gene symbol	Name	Nucleotide	Amino acid
RBM20	RNA binding motif protein 20	1906C>A	Arg636Ser
GRN	Progranulin	1477C>T	Arg493Stop
ATP7B	ATPase, Cu^{++}transporting, beta polypeptide	2333G>T	Arg778Leu
APOE	Apolipoprotein E	rs429358T>C	Cys112Arg
