# Supplementary material for: Fusion of histone variants to Cas9 suppresses non-homologous end joining
Source: PLoS One. 2024 May 13;19(5):e0288578. doi: 10.1371/journal.pone.0288578 (PMC11090291; doi:10.1371/journal.pone.0288578)
Supplement: S3 Table — (PDF) [file pone.0288578.s006.pdf]

S3 Table. Oligonucleotide donor DNAs used in this study.

Name	Sequence (5'-3')
RBM20 R636S	ACAGATATGGCCCAGAAAGGCCGCGGTCTAGTAGTCCGGTGAGCCGGTCACTCTCCCCGA
GRN R493X	CGGCTGGCTACACCTGCAACGTGAAGGCTTGATCCTGCGAGAAGGAAGTGGTCTCTGCCC
ATP7B R778L	CATGCTCTTTGTGTTCATTGCCCTGGGCCTGTGGCTGGAACACTTGGCAAAGGTAACAGC
APOE 3to4+SM	CGGCTGTCCAAGGAGCTGCAGGCGGCGCAGGCCCGGCTGGGCGCGGACATGGAGGACGTGCGCGGCCGACTGGTGCAGTACCGCGGCGAGGTGCAGGCCATGCTCGGCCAGAGCACCGAGGAGCTGCGGGTGCGCCTCGC

The targeted single nucleotide substitutions are single underlined.

The silent mutation to prevent re-cleavage by gRNA is double underlined.
