# Supplementary material for: Fusion of histone variants to Cas9 suppresses non-homologous end joining
Source: PLoS One. 2024 May 13;19(5):e0288578. doi: 10.1371/journal.pone.0288578 (PMC11090291; doi:10.1371/journal.pone.0288578)
Supplement: S4 Table — (PDF) [file pone.0288578.s007.pdf]

S4 Table. gRNAs used in this study.

Name	Sequence (5'-3')
RBM20-2 gRNA	GGTCT**C**GTAGTCCGGTGAGCCGG
RBM20-g1 gRNA	gCTCACCGGACTAC**G**AGACCGCGG
GRN-2 gRNA	GAAGGCT**C**GATCCTGCGAGAAGG
GRN-g2 gRNA	gAGAGACCACTTCCTTCTCGCAGG
ATP7B-3 gRNA	GGGCC**G**GTGGCTGGAACACTTGG
ATP7B-g3 gRNA	gAGTGTTCCAGCCAC**C**GGCCCAGG
APOE-g1 gRNA	gCCTCGCCGCGGTACTGCACCAG**G**

Protospacer adjacent motif (PAM) sequences are underlined.

Bold letters indicate the sites of single nucleotide substitutions.

Lower case "g" letters indicate the exogeneous Guanines.
