# Supplementary material for: Fusion of histone variants to Cas9 suppresses non-homologous end joining
Source: PLoS One. 2024 May 13;19(5):e0288578. doi: 10.1371/journal.pone.0288578 (PMC11090291; doi:10.1371/journal.pone.0288578)
Supplement: S5 Table — (PDF) [file pone.0288578.s008.pdf]

S5 Table. Assay components used for digital PCR in this study.

		Sequence (5'-3')	Fluor-quencher	Final concentration
**RBM20-2 assay components**				
Primers				
	Forward	CTGTGTGTGGGTGGGGT		900 nM
	Reverse	AGGAGGTGAAGCTGGGAG		900 nM
Probes				
	Reference	TGGGAGGTGTGAAGATTCTAAATC	FAM-Zen	250 nM
	HDR	CCGCGGTCTAGTAGTCC	FAM-Zen	250 nM
	Dark	CCGCGGTCTCGTAGTCC	None. Add 3' phosphate	250 nM
	2 NHEJ	AGAGTGACCGGCTCAC	HEX-Zen	250 nM
**RBM20-g1 assay components**				
Primers				
	Forward	CTGTGTGTGGGTGGGGT		900 nM
	Reverse	AGGAGGTGAAGCTGGGAG		900 nM
Probes				
	Reference	TGGGAGGTGTGAAGATTCTAAATC	FAM-Zen	250 nM
	HDR	CCGCGGTCTAGTAGTCC	FAM-Zen	250 nM
	Dark	CCGCGGTCTCGTAGTCC	None. Add 3' phosphate	250 nM
	g1 NHEJ	AGGCCGCGGTCTCGT	HEX-Zen	250 nM
**GRN-2 and GRN-g2 assay components**				
Primers				
	Forward	CTGGATAGGGGAGCTAAG		900 nM
	Reverse	GTCTGGTTATCATGGCAG		900 nM
Probes				
	Reference	CAGGAACATAATGCCATTCTGTGC	FAM-Zen	250 nM
	HDR	CAGGATCAAGCCTTCACG	FAM-Zen	250 nM
	Dark	CAGGATCGAGCCTTCACG	None. Add 3' phosphate	250 nM
	NHEJ	CTGCGAGAAGGAAGTGGT	HEX-Zen	250 nM
**ATP7B-3 assay components**				
Primers				
	Forward	TGCTTATGTTTATTCTCTGGTCATC		900 nM
	Reverse	CCTGAAGCTGCTGTTACCTT		900 nM
Probes				
	Reference	TGGTGGTTGCTGTGGCT	FAM-Zen	250 nM
	HDR	TGGGCCTGTGGCTG	FAM-Zen	250 nM
	3 NHEJ	CCGGTGGCTGGAACACT	HEX-Zen	250 nM
**ATP7B-g3 assay components**				
Primers				
	Forward	TGCTTATGTTTATTCTCTGGTCATC		900 nM
	Reverse	CCTGAAGCTGCTGTTACCTT		900 nM
Probes				
	Reference	TGGTGGTTGCTGTGGCT	FAM-Zen	250 nM
	HDR	TGGGCCTGTGGCTG	FAM-Zen	250 nM
	g3 NHEJ	CTGGGCCGGTGGCTG	HEX-Zen	250 nM
**APOE assay components**				
Primers				
	Forward	TGGAGGAACAACTGACCC		900 nM
	Reverse	GATGCCGATGACCTGCAGAA		900 nM
Probes				
	Reference	CTGTCCAAGGAGCTGCAG	FAM-Zen	250 nM
	HDR	CGTGCGCGGCCGACTGGTGC	FAM-Zen	250 nM
	NHEJ	GAGGTGCAGGCCATGCTC	HEX-Zen	250 nM

The targeted single nucleotide substitutions are single underlined.

The silent mutation to prevent re-cleavage by gRNA is double underlined.
