# Supplementary material for: Fusion of histone variants to Cas9 suppresses non-homologous end joining
Source: PLoS One. 2024 May 13;19(5):e0288578. doi: 10.1371/journal.pone.0288578 (PMC11090291; doi:10.1371/journal.pone.0288578)
Supplement: S6 Table — (PDF) [file pone.0288578.s009.pdf]

S6 Table. Thermal cycle conditions of PCR for digital PCR

Condition for RBM20	
Step (1)	95 °C 10 min.
Step (2)	94 °C 30 sec.
Step (3)	59 °C 1 min.
Step (4)	Go to Step (2). Repeat 39 cycles.
Step (5)	98 °C 10 min.

With all the steps ramped by 2°C/s

Condition for GRN, ATP7B and APOE	
Step (1)	95 °C 10 min.
Step (2)	94 °C 30 sec.
Step (3)	58 °C 1 min.
Step (4)	72 °C 2 min.
Step (5)	Go to Step (2). Repeat 39 cycles.
Step (6)	98 °C 10 min.

With all the steps ramped by 2°C/s
