# Supplementary material for: Fusion of histone variants to Cas9 suppresses non-homologous end joining
Source: PLoS One. 2024 May 13;19(5):e0288578. doi: 10.1371/journal.pone.0288578 (PMC11090291; doi:10.1371/journal.pone.0288578)
Supplement: S7 Table — (PDF) [file pone.0288578.s010.pdf]

S7 Table. Off-target sites by using the CRISPOR web tool (http://crispor.org) (Haeussler et al., 2016) .

On-Target	Name	Off-target Sequence	chrom	start	end	strand	locus Description
RBM20-2	OTS1	TCTCCTGTAGTCCGGTGAGCAGG	chr13	82,655,643	82,655,665	+	intergenic:RP11-452B18.2-RNU6-67P
	OTS2	ACTCCCGTAGTTCGGTGAGCAGG	chr8	82,160,942	82,160,964	-	intergenic:RP11-99H20.1-RP11-653B10.1
	OTS3	GCTCCCGTTGTTCGGTGAGCAGG	chr10	115,456,916	115,456,938	+	intron:ATRNL1
RBM20-g1	OTS4	CCCACTGGACTACGAGACCGTGG	chr10	71,645,941	71,645,963	+	exon:CDH23
	OTS5	CTCACAGGACCACGAGGCCCTGG	chr5	2,261,500	2,261,522	+	intergenic:Y_RNA-LSINCT5
	OTS6	ATCACAGGACCACAAGACCGAGG	chr8	109,139,437	109,139,459	+	intergenic:TRHR-NUDCD1
GRN-2	OTS7	GTTGGGTTGATCCTGCGAGAAGG	chr15	42,736,049	42,736,071	+	exon:CDAN1
	OTS8	GAAGGCTCCGTCCTGCAAGATGG	chr1	185,956,292	185,956,314	+	intron:HMCN1
	OTS9	GATGGCTTGATCCTGGGAGATGG	chr9	136,332,920	136,332,942	+	intron:GPSM1
GRN-g2	OTS10	AGAATCCACTTCCTTCTCGCAGG	chr10	129,788,553	129,788,575	+	intergenic:RP11-109A6.3-EBF3/RP11-234G16.4
	OTS11	AGGAACCTCCTCCTTCTCGCTGG	chr14	96,056,409	96,056,431	+	intron:C14orf132
	OTS12	AGGGCCCACTTCCTTCTCACTGG	chr3	197,357,241	197,357,263	-	intergenic:DLG1-AS1-AC128709.3
ATP7B-3	OTS13	GGGATGCTGGCTGGAACACTTGG	chr4	101,779,328	101,779,350	-	intergenic:RNU6-462P-BANK1
	OTS14	AGGCAGCAGGCTGGAACACTTGG	chrX	124,242,336	124,242,358	-	intergenic:SNORA40-SH2D1A
	OTS15	GGGAAGCTGTCTGGAACACTTGG	chr19	48,154,311	48,154,333	-	intron:LIG1
ATP7B-g3	OTS16	AGGGATGCAGCCACCGGCCCAGG	chr16	80,830,487	80,830,509	-	exon:RP11-314O13.1
	OTS17	AGTTTAGCAGCCACCGGCCCTGG	chr5	50,789,089	50,789,111	+	intron:PARP8
	OTS18	GGTGAACAAGCCACCGGCCCAGG	chr11	76,667,279	76,667,301	+	intron:LRRC32
APOE-g1	OTS19	GTTGGCCGTGGTACTGCACCAGG	chr20	46,011,254	46,011,276	-	exon:MMP9
	OTS20	CCTCACCTCTGCACTGCACCAGG	chr10	35,745,478	35,745,500	-	intergenic:FZD8/MIR4683-LINC01452
	OTS21	CCTCACCCCTGCACTGCACCAGG	chr11	9,141,858	9,141,880	+	intron:DENND5A

Protospacer adjacent motif (PAM) sequences are underlined.
