# Supplementary material for: Fusion of histone variants to Cas9 suppresses non-homologous end joining
Source: PLoS One. 2024 May 13;19(5):e0288578. doi: 10.1371/journal.pone.0288578 (PMC11090291; doi:10.1371/journal.pone.0288578)
Supplement: S8 Table — (PDF) [file pone.0288578.s011.pdf]

S8 Table. Oligonucleotides for the first PCR of amplicon sequencing.

On-Target	Name	Sequence (5'-3')
RBM20-2	OTS1 Fw	TAACTTACGGAGTCGCTCTACGAAAGTTGCAGCTCTTTACTCTCGTA
	OTS1 Rv	GGATGGGATTCTTTAGGTCCTGACAGGACAAGAACTTGGAACTCACT
	OTS2 Fw	TAACTTACGGAGTCGCTCTACGCTGTTCTGTGTAATGCAATGAAATC
	OTS2 Rv	GGATGGGATTCTTTAGGTCCTGGTGGTGGGAGTTAAAAGAGCTGTA
	OTS3 Fw	TAACTTACGGAGTCGCTCTACGTTTCAGCTTCTTCATTCCTGTAGTT
	OTS3 Rv	GGATGGGATTCTTTAGGTCCTGAGAGCTGTAACACTTCATTCCACTT
RBM20-g1	OTS4 Fw	TAACTTACGGAGTCGCTCTACGTCATCATCTCCCCGACCTCCGTCCA
	OTS4 Rv	GGATGGGATTCTTTAGGTCCTGCCTGAAATCCACCCCCCACCCCACT
	OTS5 Fw	TAACTTACGGAGTCGCTCTACGAACCCGGCATCTGTGTATGT
	OTS5 Rv	GGATGGGATTCTTTAGGTCCTGCAAGGGATAAGGGGACAGTAAAG
	OTS6 Fw	TAACTTACGGAGTCGCTCTACGTCCTCGCCATCTGCGGGTCTCCCC
	OTS6 Rv	GGATGGGATTCTTTAGGTCCTGTGCGCTCGGCCAGGCCGGCACCAT
GRN-2	OTS7 Fw	TAACTTACGGAGTCGCTCTACGGGTGAGGTGAAGCAGGTCTT
	OTS7 Rv	GGATGGGATTCTTTAGGTCCTGGGAGGGGAGATGTTTGAAGATAG
	OTS8 Fw	TAACTTACGGAGTCGCTCTACGCCAGGTGATCCTCCAATTCAT
	OTS8 Rv	GGATGGGATTCTTTAGGTCCTGGTAGGAACCCTGATTTATAGCCAGT
	OTS9 Fw	TAACTTACGGAGTCGCTCTACGATGATGGTGCAAACCTGAAGTC
	OTS9 Rv	GGATGGGATTCTTTAGGTCCTGATTTTTATAGAGACACGGTCTTGCT
GRN-g2	OTS10 Fw	TAACTTACGGAGTCGCTCTACGCACCCTGTCACTCAGCAAGG
	OTS10 Rv	GGATGGGATTCTTTAGGTCCTGTAGGGGATCCCAAAGCCAAA
	OTS11 Fw	TAACTTACGGAGTCGCTCTACGCCTTGACAATACTGCTCTCACATT
	OTS11 Rv	GGATGGGATTCTTTAGGTCCTGACTCGGGATTCAAACCTAAGTCAGT
	OTS12 Fw	TAACTTACGGAGTCGCTCTACGAGTTTCAAAGGCTGGGAAGTC
	OTS12 Rv	GGATGGGATTCTTTAGGTCCTGCATGGATGAGATGAGTCCCTTATAG
ATP7B-3	OTS13 Fw	TAACTTACGGAGTCGCTCTACGACTTGGGCCATATAATTCATCAGT
	OTS13 Rv	GGATGGGATTCTTTAGGTCCTGAGATGATGTCAGGGCCTTGTG
	OTS14 Fw	TAACTTACGGAGTCGCTCTACGCCTGAAATATCCTGAGAATGCTAGT
	OTS14 Rv	GGATGGGATTCTTTAGGTCCTGAACTGTTTCCTGGCATTTCTACTT
	OTS15 Fw	TAACTTACGGAGTCGCTCTACGGCCTCTCCTGGGGCTAGTTT
	OTS15 Rv	GGATGGGATTCTTTAGGTCCTGCAATGATGTAATGTACTTGGCACAG
ATP7B-g3	OTS16 Fw	TAACTTACGGAGTCGCTCTACGAACCCATAGAAACACAAGGAAGAAG
	OTS16 Rv	GGATGGGATTCTTTAGGTCCTGAGGACCATGTTCTCTCTAAAGGTTC
	OTS17 Fw	TAACTTACGGAGTCGCTCTACGGCTCTATTGAGGGCTTCCACT
	OTS17 Rv	GGATGGGATTCTTTAGGTCCTGAGGTCCACACAGGAAATAAAGTGT
	OTS18 Fw	TAACTTACGGAGTCGCTCTACGTTACAGAAGGGAAAAAGAAGTCTCA
	OTS18 Rv	GGATGGGATTCTTTAGGTCCTGCAGCACTCACTGAGGGAGCA
APOE-g1	OTS19 Fw	TAACTTACGGAGTCGCTCTACGGAGCCCCCTCACTCACTCTC
	OTS19 Rv	GGATGGGATTCTTTAGGTCCTGCACTTCCCCTTCATCTTCGAG
	OTS20 Fw	TAACTTACGGAGTCGCTCTACGAGGTGCCCACACTCATGGAT
	OTS20 Rv	GGATGGGATTCTTTAGGTCCTGACCAGAAGGTACATTAGAATGAGGA
	OTS21 Fw	TAACTTACGGAGTCGCTCTACGCAGGGCTTCTAGGAAACCTTCTA
	OTS21 Rv	GGATGGGATTCTTTAGGTCCTGGTGCTTGGGAGCCAGGTACT

Adapter sequences are underlined.
