# Supplementary material for: Fusion of histone variants to Cas9 suppresses non-homologous end joining
Source: PLoS One. 2024 May 13;19(5):e0288578. doi: 10.1371/journal.pone.0288578 (PMC11090291; doi:10.1371/journal.pone.0288578)
Supplement: S9 Table — (PDF) [file pone.0288578.s012.pdf]

S9 Table. Oligonucleotides for the second PCR of amplicon sequencing.

	Custom index	index sequence	Custom P5 adapter sequence (5' - 3')
Cas9	P01_PE1.0 (P5)	CAAGTGTTC	AATGATACGGCGACCACCGAGATCTACACTCTTTCCCTACACGACGCTCTTCCGATCTNNNNNNCAAGTGTTCTAACTTACGGAGTCGCTCTACG
	P01_PE2.0 (P7)	CAAGTGTTC	CAAGCAGAAGACGGCATACGAGATCGGTCTCGGCATTCCTGCTGAACCGCTCTTCCGATCTNNNNNCAAGTGTTCGGATGGGATTCTTTAGGTCCTG
H2A.1-Cas9	P02_PE1.0 (P5)	AGGACATTC	AATGATACGGCGACCACCGAGATCTACACTCTTTCCCTACACGACGCTCTTCCGATCTNNNNNAGGACATTCTAACTTACGGAGTCGCTCTACG
	P01_PE2.0 (P7)	CAAGTGTTC	CAAGCAGAAGACGGCATACGAGATCGGTCTCGGCATTCCTGCTGAACCGCTCTTCCGATCTNNNNNCAAGTGTTCGGATGGGATTCTTTAGGTCCTG

Index sequences are single underlined.

Adapter sequences are double underlined.
