# Supplementary material for: Fusion of histone variants to Cas9 suppresses non-homologous end joining
Source: PLoS One. 2024 May 13;19(5):e0288578. doi: 10.1371/journal.pone.0288578 (PMC11090291; doi:10.1371/journal.pone.0288578)
Supplement: S10 Table — (PDF) [file pone.0288578.s013.pdf]

S10 Table. Reagent composition and thermal cycle conditions of PCR for preparation of libraries for amplicon sequencing.

The first PCR

Reagent	Amount
Water	0.3 µL
2xPCR Buffer KOD FX	5.0 µL
2 mM dNTPs	2.0 µL
10 uM Forward Primer (Gene specific with PS1.0)	0.3 µL
10 uM Reverse Primer (Gene specific with PS2.0)	0.3 µL
5M Betaine	1.0 µL
KOD FX enzyme	0.1 µL
Genomic DNA (10-50 ng/uL)	1.0 µL
Total	10.0 µL

Condition for the first PCR	
Step (1)	95 °C 2 min.
Step (2)	95 °C 30 sec.
Step (3)	60 °C 30 sec.
Step (4)	72 °C 30 sec.
Step (5)	Go to Step (2). Repeat 29 cycles.
Step (6)	72 °C 3 min.

The second PCR

Reagent	Amount
Water	1.8 µL
2xPCR Buffer KOD FX	5.0 µL
2 mM dNTPs	2.0 µL
10 uM Forward Barcoding Primer	0.3 µL
10 uM Reverse Barcoding Primer	0.3 µL
KOD FX enzyme	0.1 µL
1st PCR product (10x dilution by MilliQ-water)	0.5 µL
Total	10.0 µL

Condition for the second PCR	
Step (1)	98 °C 2 min.
Step (2)	98 °C 30 sec.
Step (3)	57 °C 30 sec.
Step (4)	72 °C 1 min.
Step (5)	Go to Step (2). Repeat 14 cycles.
Step (6)	72 °C 3 min.
