# Supplementary material for: Fusion of histone variants to Cas9 suppresses non-homologous end joining
Source: PLoS One. 2024 May 13;19(5):e0288578. doi: 10.1371/journal.pone.0288578 (PMC11090291; doi:10.1371/journal.pone.0288578)
Supplement: S11 Table — (PDF) [file pone.0288578.s014.pdf]

S11 Table. Digital PCR raw data of Fig 1C.

Sample Name	gRNA	HDR frequency (%)	HDR average frequency (%)	HDR S.E. (%)	NHEJ frequency (%)	NHEJ average frequency (%)	NHEJ S.E. (%)	HDR / NHEJ	HDR / NHEJ average	HDR / NHEJ S.E.	Fold increase compared to Cas9
Cas9	RBM20-2	2.537	2.399	0.2631	42.14	41.72	1.549	0.0602	0.05719	0.004328	
Cas9	RBM20-2	2.771			44.18			0.06272			
Cas9	RBM20-2	1.891			38.86			0.04866			
N-GS	RBM20-2	2.517	2.421	0.113	30.2	29.25	0.5076	0.08334	0.0828	0.004108	1.447
N-GS	RBM20-2	2.196			29.11			0.07543			
N-GS	RBM20-2	2.551			28.46			0.08963			
N-GS3	RBM20-2	2.253	2.359	0.1616	26.23	28.86	1.343	0.08589	0.08185	0.004783	1.431
N-GS3	RBM20-2	2.677			30.65			0.08734			
N-GS3	RBM20-2	2.148			29.7			0.07232			
N-GS5	RBM20-2	2.571	2.39	0.1816	33.42	31.69	1.401	0.07692	0.07518	0.002596	1.314
N-GS5	RBM20-2	2.573			32.75			0.07856			
N-GS5	RBM20-2	2.027			28.92			0.07008			
C-GS	RBM20-2	2.968	2.797	0.1696	37.96	37.26	0.4883	0.07818	0.07498	0.003664	1.311
C-GS	RBM20-2	2.966			37.5			0.07909			
C-GS	RBM20-2	2.458			36.32			0.06767			
C-GS3	RBM20-2	3.57	3.085	0.2985	37.67	36.14	3.601	0.09477	0.08579	0.005489	1.5
C-GS3	RBM20-2	3.145			41.47			0.07583			
C-GS3	RBM20-2	2.541			29.28			0.08678			
C-GS5	RBM20-2	2.383	1.796	0.3108	35.99	34.94	0.7261	0.06621	0.05128	0.0083	0.8966
C-GS5	RBM20-2	1.681			33.55			0.0501			
C-GS5	RBM20-2	1.325			35.3			0.03753			
Assay background	RBM20-2	0.078	0.02928	0.02453	0.02909	0.03893	0.007415				
Assay background	RBM20-2	0.009836			0.05346						
Assay background	RBM20-2	0			0.03425						
Cas9	RBM20-g1	11.58	10.98	0.5106	20.34	21.38	0.631	0.5693	0.5147	0.02946	
Cas9	RBM20-g1	11.41			22.52			0.5066			
Cas9	RBM20-g1	9.97			21.29			0.4682			
N-GS	RBM20-g1	10.72	11.36	0.4823	20.15	21.1	0.6293	0.532	0.5382	0.006978	1.045
N-GS	RBM20-g1	11.07			20.86			0.5306			
N-GS	RBM20-g1	12.31			22.29			0.5522			
N-GS3	RBM20-g1	9.465	9.518	0.3906	15.88	15.41	0.6421	0.596	0.6178	0.01097	1.2
N-GS3	RBM20-g1	10.22			16.21			0.6304			
N-GS3	RBM20-g1	8.87			14.14			0.6272			
N-GS5	RBM20-g1	11.45	11.27	0.521	21.57	19.35	1.127	0.5308	0.5853	0.03457	1.137
N-GS5	RBM20-g1	12.08			18.6			0.6494			
N-GS5	RBM20-g1	10.3			17.89			0.5757			
C-GS	RBM20-g1	10.52	11.24	0.3642	22.69	21.19	1.25	0.4636	0.5358	0.04705	1.04
C-GS	RBM20-g1	11.68			18.71			0.6242			
C-GS	RBM20-g1	11.53			22.18			0.5198			
C-GS3	RBM20-g1	10.17	10.59	0.2453	20.21	20.82	0.4088	0.5032	0.5085	0.002741	0.9879
C-GS3	RBM20-g1	11.02			21.6			0.5101			
C-GS3	RBM20-g1	10.59			20.67			0.5123			
C-GS5	RBM20-g1	4.814	8.153	1.705	8.986	14.45	2.857	0.5357	0.56	0.01402	1.088
C-GS5	RBM20-g1	10.43			18.62			0.5601			
C-GS5	RBM20-g1	9.215			15.77			0.5843			
Assay background	RBM20-g1	0.09628	0.0498	0.02479	0.06975	0.06542	0.018				
Assay background	RBM20-g1	0.01159			0.0323						
Assay background	RBM20-g1	0.04154			0.09421						
Cas9	GRN-2	0.5704	0.7477	0.09119	25.41	22.52	2.911	0.02244	0.03486	0.007343	
Cas9	GRN-2	0.8734			25.46			0.0343			
Cas9	GRN-2	0.7993			16.7			0.04786			
N-GS	GRN-2	0.6007	0.8582	0.1411	13.97	16.48	1.337	0.04299	0.05131	0.004549	1.471
N-GS	GRN-2	1.087			18.53			0.05866			
N-GS	GRN-2	0.8869			16.96			0.05229			
N-GS3	GRN-2	1.125	0.9822	0.08341	17.66	18.6	0.8324	0.0637	0.05303	0.00536	1.521
N-GS3	GRN-2	0.8361			17.88			0.04676			
N-GS3	GRN-2	0.9855			20.26			0.04864			
N-GS5	GRN-2	0.4192	0.7318	0.1709	16.88	19.21	2.534	0.02483	0.03764	0.006572	1.079
N-GS5	GRN-2	1.008			24.28			0.04151			
N-GS5	GRN-2	0.7683			16.49			0.04659			
C-GS	GRN-2	1.201	0.9511	0.1403	17.07	16.44	0.4638	0.07035	0.0578	0.008057	1.658
C-GS	GRN-2	0.9369			15.54			0.06028			
C-GS	GRN-2	0.7156			16.73			0.04277			
C-GS3	GRN-2	0.7197	0.8223	0.09834	16.68	16.22	0.3469	0.04314	0.05066	0.005759	1.453
C-GS3	GRN-2	1.019			16.44			0.06198			
C-GS3	GRN-2	0.7284			15.54			0.04687			

C-GS5	GRN-2	0.8745	0.9172	0.05424	19.66	19.21	0.9696	0.04448	0.04775	0.001643	1.369
C-GS5	GRN-2	1.025			20.63			0.04968			
C-GS5	GRN-2	0.8523			17.36			0.04909			
Assay background	GRN-2	0.05387	0.03691	0.01122	0.02768	0.0194	0.009738				
Assay background	GRN-2	0.04114			0.03054						
Assay background	GRN-2	0.01571			0						
Cas9	GRN-g2	0.8645	0.985	0.0683	19.42	17.97	1.058	0.04451	0.05565	0.007227	
Cas9	GRN-g2	0.9896			18.58			0.05326			
Cas9	GRN-g2	1.101			15.91			0.0692			
N-GS	GRN-g2	0.8448	0.8544	0.06035	13.67	14.06	0.2085	0.06179	0.06581	0.003962	1.182
N-GS	GRN-g2	0.755			14.14			0.05339			
N-GS	GRN-g2	0.9634			14.38			0.06699			
N-GS3	GRN-g2	0.9711	0.8567	0.06049	12.6	12.3	0.3341	0.07707	0.06954	0.003775	1.249
N-GS3	GRN-g2	0.8336			12.68			0.06574			
N-GS3	GRN-g2	0.7654			11.64			0.06575			
N-GS5	GRN-g2	1.073	1.001	0.05396	13.91	14.36	0.367	0.07713	0.0703	0.005421	1.263
N-GS5	GRN-g2	0.8955			15.09			0.05934			
N-GS5	GRN-g2	1.035			14.09			0.07345			
C-GS	GRN-g2	1.332	1.151	0.1086	17.05	17.01	1.371	0.07812	0.05606	0.009872	1.007
C-GS	GRN-g2	1.167			14.62			0.07982			
C-GS	GRN-g2	0.9567			19.37			0.04939			
C-GS3	GRN-g2	0.7505	0.858	0.1568	19.25	16.41	1.434	0.03898	0.05384	0.01303	0.9674
C-GS3	GRN-g2	0.6567			15.37			0.04272			
C-GS3	GRN-g2	1.167			14.62			0.07982			
C-GS5	GRN-g2	0.7505	0.851	0.0902	19.25	18.46	0.4988	0.03898	0.04969	0.004859	0.8929
C-GS5	GRN-g2	1.031			18.61			0.0554			
C-GS5	GRN-g2	0.7715			17.54			0.04398			
Assay background	GRN-g2	0.02714	0.03653	0.01681	0	0.03758	0.02908				
Assay background	GRN-g2	0.06918			0.01792						
Assay background	GRN-g2	0.01326			0.09481						
