# Supplementary material for: Fusion of histone variants to Cas9 suppresses non-homologous end joining
Source: PLoS One. 2024 May 13;19(5):e0288578. doi: 10.1371/journal.pone.0288578 (PMC11090291; doi:10.1371/journal.pone.0288578)
Supplement: S12 Table — (PDF) [file pone.0288578.s015.pdf]

S12 Table. Digital PCR raw data of Fig 2B.

Sample Name	gRNA	HDR frequency (%)	HDR average frequency (%)	HDR S.E. (%)	NHEJ frequency (%)	NHEJ average frequency (%)	NHEJ S.E. (%)	HDR / NHEJ	HDR / NHEJ average	HDR / NHEJ S.E.	Fold increase compared to N-GS3
N-GS3	RBM20-2	3.286	2.513	0.4354	35.16	31.81	1.871	0.09345	0.07795	0.009081	
N-GS3	RBM20-2	1.779			28.69			0.062			
N-GS3	RBM20-2	2.476			31.58			0.0784			
SD-Cas9	RBM20-2	2.235	2.521	0.3034	26.84	28.7	1.428	0.08327	0.08857	0.01262	1.136
SD-Cas9	RBM20-2	2.201			31.51			0.06985			
SD-Cas9	RBM20-2	3.128			27.76			0.1126			
SA-Cas9	RBM20-2	1.973	1.913	0.0741	27.69	26.66	0.663	0.07125	0.07189	0.003763	0.9222
SA-Cas9	RBM20-2	2.001			25.42			0.07871			
SA-Cas9	RBM20-2	1.766			26.87			0.06572			
KL-Cas9	RBM20-2	1.703	1.919	0.1807	23.77	25.64	2.424	0.07164	0.07675	0.01238	0.9846
KL-Cas9	RBM20-2	1.776			30.45			0.05832			
KL-Cas9	RBM20-2	2.278			22.7			0.1003			
KM-Cas9	RBM20-2	2.174	1.986	0.1518	34.19	33.36	0.5387	0.06358	0.05943	0.003672	0.7624
KM-Cas9	RBM20-2	1.686			32.35			0.05211			
KM-Cas9	RBM20-2	2.1			33.54			0.06261			
KA-Cas9	RBM20-2	2.104	1.984	0.07299	30.45	28.09	2.71	0.06909	0.07159	0.005218	0.9184
KA-Cas9	RBM20-2	1.852			22.69			0.08162			
KA-Cas9	RBM20-2	1.996			31.15			0.06407			
KL_SD-Cas9	RBM20-2	1.288	1.613	0.2035	20.74	22.76	3.575	0.0621	0.07224	0.007858	0.9267
KL_SD-Cas9	RBM20-2	1.564			17.83			0.08771			
KL_SD-Cas9	RBM20-2	1.988			29.71			0.06691			
KM_SD-Cas9	RBM20-2	1.705	1.998	0.1591	26.37	27.34	0.5461	0.06465	0.07291	0.004402	0.9353
KM_SD-Cas9	RBM20-2	2.038			27.39			0.0744			
KM_SD-Cas9	RBM20-2	2.252			28.26			0.07968			
KA_SD-Cas9	RBM20-2	1.593	1.931	0.3345	22.14	23.04	1.686	0.07195	0.08271	0.008205	1.061
KA_SD-Cas9	RBM20-2	1.6			20.68			0.07736			
KA_SD-Cas9	RBM20-2	2.6			26.31			0.09882			
N-GS3	RBM20-g1	15.22	15.7	0.2623	24.25	23.22	0.5141	0.6276	0.6773	0.02511	
N-GS3	RBM20-g1	16.12			22.76			0.7082			
N-GS3	RBM20-g1	15.78			22.66			0.6963			
SD-Cas9	RBM20-g1	12.66	15.18	1.331	25.77	24.79	0.7628	0.4912	0.6147	0.06177	0.9075
SD-Cas9	RBM20-g1	15.71			23.29			0.6745			
SD-Cas9	RBM20-g1	17.18			25.32			0.6785			
SA-Cas9	RBM20-g1	13.81	14.44	0.319	22.34	22.12	0.7871	0.6181	0.6547	0.03185	0.9666
SA-Cas9	RBM20-g1	14.67			23.36			0.6279			
SA-Cas9	RBM20-g1	14.84			20.66			0.7182			
KL-Cas9	RBM20-g1	16.01	15.48	0.5333	24.38	21.95	1.254	0.6566	0.7077	0.0278	1.044
KL-Cas9	RBM20-g1	14.42			20.18			0.7145			
KL-Cas9	RBM20-g1	16.03			21.31			0.7522			
KM-Cas9	RBM20-g1	13.45	14.64	0.6558	22.92	21.81	0.8038	0.5868	0.674	0.04416	0.9951
KM-Cas9	RBM20-g1	15.71			22.27			0.7054			
KM-Cas9	RBM20-g1	14.78			20.25			0.7298			
KA-Cas9	RBM20-g1	13.81	15.96	1.349	24.57	24.02	0.295	0.562	0.6655	0.06031	0.9825
KA-Cas9	RBM20-g1	15.64			23.56			0.6638			
KA-Cas9	RBM20-g1	18.45			23.93			0.7709			
KL_SD-Cas9	RBM20-g1	13.76	14.04	0.2833	20.93	20.43	0.6013	0.6574	0.6878	0.01673	1.015
KL_SD-Cas9	RBM20-g1	14.61			21.14			0.6911			
KL_SD-Cas9	RBM20-g1	13.76			19.24			0.7151			
KM_SD-Cas9	RBM20-g1	15.09	15.43	0.1809	27.94	24.62	1.687	0.54	0.6332	0.04718	0.9348
KM_SD-Cas9	RBM20-g1	15.52			22.41			0.6925			
KM_SD-Cas9	RBM20-g1	15.7			23.53			0.6672			
KA_SD-Cas9	RBM20-g1	12.12	13.8	0.8971	20.9	21.9	1.441	0.5799	0.6355	0.06042	0.9382
KA_SD-Cas9	RBM20-g1	15.18			20.07			0.7563			
KA_SD-Cas9	RBM20-g1	14.12			24.75			0.5705			
N-GS3	GRN-2	1.519	1.92	0.2474	34.34	34.91	0.8217	0.04423	0.0548	0.005979	
N-GS3	GRN-2	1.871			33.86			0.05525			
N-GS3	GRN-2	2.372			36.53			0.06493			
SD-Cas9	GRN-2	1.913	1.9	0.1268	36.12	38.84	2.746	0.05296	0.049	0.002013	0.8941
SD-Cas9	GRN-2	1.674			36.08			0.04639			
SD-Cas9	GRN-2	2.113			44.34			0.04765			
SA-Cas9	GRN-2	2.496	2.05	0.2281	36.56	36.57	0.906	0.06827	0.05594	0.006167	1.02
SA-Cas9	GRN-2	1.757			35.01			0.05018			
SA-Cas9	GRN-2	1.884			38.15			0.04938			
KL-Cas9	GRN-2	1.186	1.91	0.3797	37.85	41.58	2.484	0.03133	0.04526	0.006998	0.8259
KL-Cas9	GRN-2	2.47			46.29			0.05335			
KL-Cas9	GRN-2	2.076			40.61			0.05112			

KM-Cas9	GRN-2	1.746	1.749	0.04708	46.44	41.96	3.688	0.03759	0.04222	0.003131	0.7704
KM-Cas9	GRN-2	1.833			44.81			0.0409			
KM-Cas9	GRN-2	1.67			34.65			0.04819			
KA-Cas9	GRN-2	1.8	2.26	0.3273	41.74	42.13	1.476	0.04312	0.05335	0.006185	0.9735
KA-Cas9	GRN-2	2.894			44.87			0.06449			
KA-Cas9	GRN-2	2.088			39.8			0.05246			
KL_SD-Cas9	GRN-2	1.616	1.792	0.1171	34.52	35.58	1.04	0.04681	0.05026	0.001926	0.9171
KL_SD-Cas9	GRN-2	1.746			34.56			0.05052			
KL_SD-Cas9	GRN-2	2.014			37.66			0.05347			
KM_SD-Cas9	GRN-2	2.274	1.894	0.2094	45.08	43.72	0.8496	0.05044	0.04327	0.004392	0.7895
KM_SD-Cas9	GRN-2	1.551			43.94			0.03529			
KM_SD-Cas9	GRN-2	1.859			42.16			0.04409			
KA_SD-Cas9	GRN-2	1.997	1.977	0.0998	32.18	34.38	1.236	0.06205	0.05757	0.002945	1.05
KA_SD-Cas9	GRN-2	1.795			34.5			0.05202			
KA_SD-Cas9	GRN-2	2.139			36.46			0.05866			
N-GS3	GRN-g2	0.8237	0.7192	0.05308	12.63	11.89	1.001	0.06521	0.06121	0.005943	
N-GS3	GRN-g2	0.6507			13.14			0.04952			
N-GS3	GRN-g2	0.6832			9.913			0.06891			
SD-Cas9	GRN-g2	1.037	0.9579	0.06089	19.43	17.79	0.9586	0.05337	0.05412	0.00434	0.8841
SD-Cas9	GRN-g2	0.9987			16.11			0.06199			
SD-Cas9	GRN-g2	0.8382			17.83			0.04701			
SA-Cas9	GRN-g2	0.7209	0.7361	0.06622	15.35	13.45	0.9532	0.04696	0.05537	0.006947	0.9045
SA-Cas9	GRN-g2	0.6297			12.59			0.05001			
SA-Cas9	GRN-g2	0.8576			12.4			0.06916			
KL-Cas9	GRN-g2	0.461	0.7684	0.1553	11.48	13.74	1.191	0.04015	0.05486	0.007965	0.8962
KL-Cas9	GRN-g2	0.8836			15.52			0.05693			
KL-Cas9	GRN-g2	0.9608			14.23			0.06751			
KM-Cas9	GRN-g2	0.8657	0.9329	0.03516	17.47	16.98	0.3051	0.04955	0.05504	0.003016	0.8991
KM-Cas9	GRN-g2	0.9487			17.05			0.05564			
KM-Cas9	GRN-g2	0.9844			16.42			0.05995			
KA-Cas9	GRN-g2	1	1.226	0.1178	21.39	22.56	0.7159	0.04675	0.05416	0.003724	0.8848
KA-Cas9	GRN-g2	1.284			22.43			0.05724			
KA-Cas9	GRN-g2	1.396			23.86			0.0585			
KL_SD-Cas9	GRN-g2	0.7715	0.8417	0.04425	17.54	16.81	1.075	0.04398	0.05039	0.003611	0.8232
KL_SD-Cas9	GRN-g2	0.9235			18.21			0.05071			
KL_SD-Cas9	GRN-g2	0.8303			14.7			0.05648			
KM_SD-Cas9	GRN-g2	1.097	1.029	0.03914	17.27	17.39	0.6724	0.06352	0.05931	0.002348	0.9689
KM_SD-Cas9	GRN-g2	0.9614			16.29			0.05901			
KM_SD-Cas9	GRN-g2	1.031			18.61			0.0554			
KA_SD-Cas9	GRN-g2	0.5607	0.5462	0.1171	11.32	11.36	1.457	0.04953	0.04693	0.00462	0.7667
KA_SD-Cas9	GRN-g2	0.7416			13.91			0.05331			
KA_SD-Cas9	GRN-g2	0.3364			8.863			0.03795			
