# Supplementary material for: Fusion of histone variants to Cas9 suppresses non-homologous end joining
Source: PLoS One. 2024 May 13;19(5):e0288578. doi: 10.1371/journal.pone.0288578 (PMC11090291; doi:10.1371/journal.pone.0288578)
Supplement: S13 Table — (PDF) [file pone.0288578.s016.pdf]

S13 Table. Digital PCR raw data of Fig 3B.

Sample Name	gRNA	HDR frequency (%)	HDR average frequency (%)	HDR S.E. (%)	NHEJ frequency (%)	NHEJ average frequency (%)	NHEJ S.E. (%)	HDR / NHEJ	HDR / NHEJ average	HDR / NHEJ S.E.	Fold increase compared to N-GS3
N-GS3	RBM20-2	3.286	2.513	0.4354	35.16	31.81	1.871	0.09345	0.07795	0.009081	
N-GS3	RBM20-2	1.779			28.69			0.062			
N-GS3	RBM20-2	2.476			31.58			0.0784			
KQ-Cas9	RBM20-2	1.883	2.31	0.2989	30.07	29.42	1.313	0.06262	0.07837	0.008586	1.005
KQ-Cas9	RBM20-2	2.161			26.9			0.08033			
KQ-Cas9	RBM20-2	2.886			31.31			0.09217			
KR-Cas9	RBM20-2	1.905	2.238	0.1881	19.29	24.38	2.549	0.09875	0.09243	0.00436	1.185
KR-Cas9	RBM20-2	2.255			26.82			0.08407			
KR-Cas9	RBM20-2	2.556			27.05			0.09449			
N-GS3	RBM20-g1	15.22	15.7	0.2623	24.25	23.22	0.5141	0.6276	0.6773	0.02511	
N-GS3	RBM20-g1	16.12			22.76			0.7082			
N-GS3	RBM20-g1	15.78			22.66			0.6963			
KQ-Cas9	RBM20-g1	15.06	15.73	0.4504	23.27	23.37	1.103	0.6471	0.6775	0.0478	1
KQ-Cas9	RBM20-g1	15.56			25.33			0.6142			
KQ-Cas9	RBM20-g1	16.59			21.51			0.7712			
KR-Cas9	RBM20-g1	12.94	15.35	1.226	19.53	20.4	0.4463	0.6625	0.7505	0.04633	1.108
KR-Cas9	RBM20-g1	16.16			21			0.7695			
KR-Cas9	RBM20-g1	16.95			20.68			0.8196			
N-GS3	GRN-2	1.519	1.92	0.2474	34.34	34.91	0.8217	0.04423	0.0548	0.005979	
N-GS3	GRN-2	1.871			33.86			0.05525			
N-GS3	GRN-2	2.372			36.53			0.06493			
KQ-Cas9	GRN-2	2.73	2.252	0.241	43.73	41.01	1.373	0.06242	0.05465	0.004016	0.9972
KQ-Cas9	GRN-2	1.959			39.98			0.04899			
KQ-Cas9	GRN-2	2.067			39.32			0.05256			
KR-Cas9	GRN-2	1.951	1.808	0.07979	36.82	31.54	2.691	0.05298	0.05781	0.00339	1.054
KR-Cas9	GRN-2	1.8			27.97			0.06435			
KR-Cas9	GRN-2	1.675			29.85			0.05611			
N-GS3	GRN-g2	0.8237	0.7192	0.05308	12.63	11.89	1.001	0.06521	0.06121	0.005943	
N-GS3	GRN-g2	0.6507			13.14			0.04952			
N-GS3	GRN-g2	0.6832			9.913			0.06891			
KQ-Cas9	GRN-g2	0.9502	1.027	0.04021	17.29	19.19	2.152	0.05495	0.05469	0.005821	0.8934
KQ-Cas9	GRN-g2	1.045			23.49			0.04448			
KQ-Cas9	GRN-g2	1.086			16.8			0.06464			
KR-Cas9	GRN-g2	0.7316	0.5878	0.07829	13.46	11.51	1.044	0.05435	0.05108	0.005016	0.8345
KR-Cas9	GRN-g2	0.5697			9.88			0.05766			
KR-Cas9	GRN-g2	0.4622			11.21			0.04123			
