# Supplementary material for: Fusion of histone variants to Cas9 suppresses non-homologous end joining
Source: PLoS One. 2024 May 13;19(5):e0288578. doi: 10.1371/journal.pone.0288578 (PMC11090291; doi:10.1371/journal.pone.0288578)
Supplement: S14 Table — (PDF) [file pone.0288578.s017.pdf]

S14 Table. Digital PCR raw data of Figs 4B and 4D.

Sample Name	gRNA	HDR frequency (%)	HDR average frequency (%)	HDR S.E. (%)	NHEJ frequency (%)	NHEJ average frequency (%)	NHEJ S.E. (%)	HDR / NHEJ	HDR / NHEJ average	HDR / NHEJ S.E.	Fold increase compared to N-GS3
H2A.X (N-GS3)	RBM20-2	3.286	2.513	0.4354	35.16	31.81	1.871	0.09345	0.07795	0.009081	
H2A.X (N-GS3)	RBM20-2	1.779			28.69			0.062			
H2A.X (N-GS3)	RBM20-2	2.476			31.58			0.0784			
H2A-Cas9	RBM20-2	2.722	2.244	0.2807	30.96	29.1	2.509	0.08791	0.07685	0.005567	0.9858
H2A-Cas9	RBM20-2	2.261			32.22			0.07017			
H2A-Cas9	RBM20-2	1.75			24.14			0.07249			
H2A.1-Cas9	RBM20-2	1.979	1.969	0.1149	22.51	20.39	1.122	0.08791	0.09689	0.006008	1.242
H2A.1-Cas9	RBM20-2	2.164			19.97			0.1083			
H2A.1-Cas9	RBM20-2	1.766			18.69			0.09448			
H2A.2-Cas9	RBM20-2	2.307	2.191	0.07911	27.13	24.02	1.97	0.08503	0.09192	0.004397	1.179
H2A.2-Cas9	RBM20-2	2.04			20.37			0.1001			
H2A.2-Cas9	RBM20-2	2.227			24.57			0.09063			
H2A.L-Cas9	RBM20-2	2.193	1.775	0.2172	29.11	25.14	2.14	0.07533	0.0702	0.002578	0.9005
H2A.L-Cas9	RBM20-2	1.462			21.77			0.06715			
H2A.L-Cas9	RBM20-2	1.672			24.54			0.06813			
H2A.J-Cas9	RBM20-2	1.501	1.682	0.09068	18.6	20.84	1.546	0.08069	0.08108	0.003867	1.04
H2A.J-Cas9	RBM20-2	1.776			23.81			0.07459			
H2A.J-Cas9	RBM20-2	1.77			20.12			0.08797			
H2A.Z-Cas9	RBM20-2	1.686	1.71	0.1422	19.18	21.58	2.077	0.0879	0.0796	0.004192	1.021
H2A.Z-Cas9	RBM20-2	1.968			25.72			0.07651			
H2A.Z-Cas9	RBM20-2	1.477			19.85			0.0744			
macroH2A.1-Cas9	RBM20-2	2.179	2.15	0.01567	28.97	27.87	2.015	0.07521	0.078	0.006033	1
macroH2A.1-Cas9	RBM20-2	2.125			30.69			0.06924			
macroH2A.1-Cas9	RBM20-2	2.147			23.97			0.08957			
H2A.B-Cas9	RBM20-2	2.763	2.5	0.1476	25.16	29.16	2.626	0.1098	0.08751	0.01132	1.122
H2A.B-Cas9	RBM20-2	2.252			28.21			0.07982			
H2A.B-Cas9	RBM20-2	2.487			34.11			0.07291			
H2B-Cas9	RBM20-2	1.917	2.081	0.4062	28.28	28.36	0.1729	0.06778	0.07321	0.01382	0.9391
H2B-Cas9	RBM20-2	2.853			28.7			0.0994			
H2B-Cas9	RBM20-2	1.475			28.12			0.05245			
H2A.X (N-GS3)	RBM20-g1	15.22	15.7	0.2623	24.25	23.22	0.5141	0.6276	0.6773	0.02511	
H2A.X (N-GS3)	RBM20-g1	16.12			22.76			0.7082			
H2A.X (N-GS3)	RBM20-g1	15.78			22.66			0.6963			
H2A-Cas9	RBM20-g1	14.75	14.76	0.5571	24.5	23.68	0.8671	0.602	0.6233	0.01112	0.9202
H2A-Cas9	RBM20-g1	13.8			21.95			0.6287			
H2A-Cas9	RBM20-g1	15.73			24.6			0.6394			
H2A.1-Cas9	RBM20-g1	11.38	11.44	0.4025	18.92	17.2	0.8927	0.6014	0.668	0.03616	0.9862
H2A.1-Cas9	RBM20-g1	12.17			16.77			0.7257			
H2A.1-Cas9	RBM20-g1	10.78			15.92			0.6771			
H2A.2-Cas9	RBM20-g1	13.22	15.87	1.339	22.11	22.93	0.4524	0.5979	0.6905	0.04852	1.019
H2A.2-Cas9	RBM20-g1	17.54			23.02			0.7619			
H2A.2-Cas9	RBM20-g1	16.85			23.67			0.7118			
H2A.L-Cas9	RBM20-g1	14.47	15.19	0.4305	27.12	24.06	1.579	0.5335	0.639	0.05727	0.9434
H2A.L-Cas9	RBM20-g1	15.16			23.21			0.6531			
H2A.L-Cas9	RBM20-g1	15.96			21.85			0.7304			
H2A.J-Cas9	RBM20-g1	12.59	11.91	0.3926	15.07	13.36	0.8731	0.8354	0.8964	0.04267	1.323
H2A.J-Cas9	RBM20-g1	11.23			12.83			0.8752			
H2A.J-Cas9	RBM20-g1	11.93			12.19			0.9786			
H2A.Z-Cas9	RBM20-g1	12.39	10.66	1.033	20.27	16.82	1.737	0.6112	0.6354	0.03043	0.9381
H2A.Z-Cas9	RBM20-g1	10.78			15.49			0.6959			
H2A.Z-Cas9	RBM20-g1	8.815			14.71			0.5992			
macroH2A.1-Cas9	RBM20-g1	15.26	15.26	0.3608	20.14	20.96	1.291	0.7576	0.7312	0.02759	1.079
macroH2A.1-Cas9	RBM20-g1	14.64			19.26			0.7601			
macroH2A.1-Cas9	RBM20-g1	15.89			23.5			0.6761			
H2A.B-Cas9	RBM20-g1	12.87	14.27	0.7028	18.32	20.26	0.9984	0.7025	0.7043	0.004963	1.039
H2A.B-Cas9	RBM20-g1	14.86			20.82			0.7137			
H2A.B-Cas9	RBM20-g1	15.08			21.64			0.6968			
H2B-Cas9	RBM20-g1	10.35	9.86	0.3588	20.25	18.7	0.892	0.5111	0.5291	0.02947	0.7811
H2B-Cas9	RBM20-g1	10.07			17.16			0.5868			
H2B-Cas9	RBM20-g1	9.161			18.71			0.4896			
H2A.X (N-GS3)	GRN-2	2.002	2.082	0.15	34.34	34.91	0.8218	0.05829	0.05949	0.002858	
H2A.X (N-GS3)	GRN-2	1.871			33.86			0.05525			
H2A.X (N-GS3)	GRN-2	2.372			36.53			0.06493			
H2A-Cas9	GRN-2	1.193	1.631	0.2201	41.04	43.22	2.074	0.02906	0.03769	0.004803	0.6335
H2A-Cas9	GRN-2	1.818			47.37			0.03837			
H2A-Cas9	GRN-2	1.884			41.26			0.04566			
H2A.1-Cas9	GRN-2	1.572	1.469	0.05507	22.92	23.56	2.592	0.06858	0.06401	0.007787	1.075
H2A.1-Cas9	GRN-2	1.45			19.43			0.07462			
H2A.1-Cas9	GRN-2	1.384			28.34			0.04883			
H2A.2-Cas9	GRN-2	1.94	1.98	0.06493	41.8	41.6	3.067	0.04641	0.0479	0.002232	0.8051
H2A.2-Cas9	GRN-2	2.107			46.82			0.045			
H2A.2-Cas9	GRN-2	1.893			36.2			0.05229			
H2A.L-Cas9	GRN-2	1.209	1.567	0.2574	35.79	36.89	1.572	0.03378	0.04211	0.0052	0.7078
H2A.L-Cas9	GRN-2	2.067			40			0.05167			
H2A.L-Cas9	GRN-2	1.427			34.9			0.04088			
H2A.J-Cas9	GRN-2	0.5467	0.7142	0.08839	8.584	11.69	1.58	0.06368	0.06132	0.001474	1.03
H2A.J-Cas9	GRN-2	0.8469			13.73			0.06168			
H2A.J-Cas9	GRN-2	0.7491			12.78			0.05861			
H2A.Z-Cas9	GRN-2	1.42	1.249	0.161	40.03	34.02	3.151	0.03547	0.03664	0.003317	0.6159
H2A.Z-Cas9	GRN-2	1.401			32.66			0.04289			
H2A.Z-Cas9	GRN-2	0.9276			29.37			0.03158			
macroH2A.1-Cas9	GRN-2	1.73	1.636	0.04789	29.92	28.09	1.182	0.05782	0.05834	0.00198	0.9806

macroH2A.1-Cas9	GRN-2	1.605			25.88			0.06201			
macroH2A.1-Cas9	GRN-2	1.573			28.49			0.05521			
H2A.B-Cas9	GRN-2	1.433	1.548	0.1349	39.11	40.2	0.6666	0.03664	0.03854	0.0035	0.6478
H2A.B-Cas9	GRN-2	1.394			41.41			0.03366			
H2A.B-Cas9	GRN-2	1.817			40.08			0.04533			
H2B-Cas9	GRN-2	0.7497	0.783	0.0544	26.7	22.56	2.203	0.02807	0.03566	0.005507	0.5994
H2B-Cas9	GRN-2	0.71			21.81			0.03255			
H2B-Cas9	GRN-2	0.8894			19.18			0.04637			
H2A.X (N-GS3)	GRN-g2	0.8237	0.7192	0.05308	12.63	11.89	1.001	0.06521	0.06121	0.005943	
H2A.X (N-GS3)	GRN-g2	0.6507			13.14			0.04952			
H2A.X (N-GS3)	GRN-g2	0.6832			9.913			0.06891			
H2A-Cas9	GRN-g2	0.725	0.8211	0.06202	19.3	19.88	0.8707	0.03756	0.04121	0.001838	0.6732
H2A-Cas9	GRN-g2	0.8014			18.76			0.04271			
H2A-Cas9	GRN-g2	0.9371			21.6			0.04338			
H2A.1-Cas9	GRN-g2	0.7607	0.5569	0.1035	12.28	9.617	1.347	0.06194	0.0572	0.00251	0.9344
H2A.1-Cas9	GRN-g2	0.4234			7.929			0.05339			
H2A.1-Cas9	GRN-g2	0.4866			8.644			0.05629			
H2A.2-Cas9	GRN-g2	0.8133	0.9271	0.06784	19.08	19.3	0.8043	0.04262	0.04833	0.004917	0.7895
H2A.2-Cas9	GRN-g2	1.048			18.03			0.05812			
H2A.2-Cas9	GRN-g2	0.92			20.79			0.04425			
H2A.L-Cas9	GRN-g2	0.7	0.9111	0.132	21.08	22.17	0.5575	0.0332	0.04086	0.00504	0.6675
H2A.L-Cas9	GRN-g2	0.8795			22.53			0.03903			
H2A.L-Cas9	GRN-g2	1.154			22.91			0.05037			
H2A.J-Cas9	GRN-g2	0.8929	0.8779	0.07114	16.78	13.95	1.547	0.05321	0.06379	0.00572	1.042
H2A.J-Cas9	GRN-g2	0.993			13.63			0.07285			
H2A.J-Cas9	GRN-g2	0.7479			11.45			0.06531			
H2A.Z-Cas9	GRN-g2	0.5561	0.5966	0.09014	9.327	10.54	0.6547	0.05962	0.05644	0.006882	0.922
H2A.Z-Cas9	GRN-g2	0.4647			10.74			0.04326			
H2A.Z-Cas9	GRN-g2	0.769			11.57			0.06646			
macroH2A.1-Cas9	GRN-g2	1.086	1.064	0.01827	16.3	16.58	0.1809	0.06662	0.06419	0.001298	1.048
macroH2A.1-Cas9	GRN-g2	1.079			16.92			0.06377			
macroH2A.1-Cas9	GRN-g2	1.028			16.53			0.06218			
H2A.B-Cas9	GRN-g2	1.449	1.209	0.1387	21.66	16.32	2.849	0.06689	0.07556	0.004406	1.234
H2A.B-Cas9	GRN-g2	1.21			15.4			0.07857			
H2A.B-Cas9	GRN-g2	0.9684			11.92			0.08124			
H2B-Cas9	GRN-g2	0.6077	0.7299	0.08307	14.26	14.9	0.8962	0.04261	0.04875	0.003185	0.7964
H2B-Cas9	GRN-g2	0.8885			16.67			0.05329			
H2B-Cas9	GRN-g2	0.6935			13.77			0.05036			
H3-Cas9	RBM20-2	3.159	2.982	0.0885	33.14	33.03	0.5598	0.09532	0.09031	0.002895	0.26426648
H3-Cas9	RBM20-2	2.892			32.01			0.09034			
H3-Cas9	RBM20-2	2.895			33.94			0.08529			
H3.1-Cas9	RBM20-2	2.958	2.284	0.3452	35.74	29.77	3.025	0.08276	0.07599	0.003689	0.85152408
H3.1-Cas9	RBM20-2	2.079			27.66			0.07516			
H3.1-Cas9	RBM20-2	1.816			25.92			0.07006			
H3.2-Cas9	RBM20-2	2.165	1.948	0.1651	34.5	26.73	3.93	0.06275	0.0744	0.006697	0.76920879
H3.2-Cas9	RBM20-2	2.056			23.92			0.08595			
H3.2-Cas9	RBM20-2	1.624			21.79			0.07452			
H3.3-Cas9	RBM20-2	1.409	1.487	0.1576	16.22	18.07	0.9293	0.08686	0.0826	0.008682	0.72970458
H3.3-Cas9	RBM20-2	1.262			19.15			0.0659			
H3.3-Cas9	RBM20-2	1.791			18.84			0.09506			
H3-Cas9	RBM20-g1	11.03	11.23	0.21	22.39	19.87	1.478	0.4926	0.5711	0.04226	0.09682979
H3-Cas9	RBM20-g1	11.65			19.97			0.5833			
H3-Cas9	RBM20-g1	11.01			17.27			0.6375			
H3.1-Cas9	RBM20-g1	8.161	8.747	0.309	18.72	16.67	1.025	0.4359	0.5305	0.0478	0.05303082
H3.1-Cas9	RBM20-g1	9.21			15.62			0.5896			
H3.1-Cas9	RBM20-g1	8.871			15.67			0.5661			
H3.2-Cas9	RBM20-g1	8.357	8.14	0.1894	16.16	15.64	0.3031	0.5171	0.5204	0.005095	0.00360139
H3.2-Cas9	RBM20-g1	7.763			15.11			0.5137			
H3.2-Cas9	RBM20-g1	8.302			15.65			0.5304			
H3.3-Cas9	RBM20-g1	11.65	8.607	1.521	19.97	14.68	2.652	0.5833	0.5872	0.01196	0.03165634
H3.3-Cas9	RBM20-g1	7.102			11.65			0.6096			
H3.3-Cas9	RBM20-g1	7.069			12.43			0.5687			
H3-Cas9	GRN-2	0.7557	1.059	0.1753	33.55	33.7	2.163	0.02252	0.03203	0.006867	0.02099862
H3-Cas9	GRN-2	1.059			37.53			0.02821			
H3-Cas9	GRN-2	1.363			30.04			0.04537			
H3.1-Cas9	GRN-2	0.9863	0.9626	0.01362	38.82	34.48	2.323	0.0254	0.0281	0.001462	0.00061367
H3.1-Cas9	GRN-2	0.9624			33.76			0.0285			
H3.1-Cas9	GRN-2	0.9391			30.87			0.03042			
H3.2-Cas9	GRN-2	0.7293	0.8212	0.04598	33.95	34.22	0.5061	0.02148	0.02399	0.001304	0.00034982
H3.2-Cas9	GRN-2	0.8681			35.2			0.02466			
H3.2-Cas9	GRN-2	0.8664			33.51			0.02585			
H3.3-Cas9	GRN-2	0.6387	0.8227	0.09899	24.62	22.57	1.296	0.02594	0.03693	0.005498	0.02196153
H3.3-Cas9	GRN-2	0.978			22.93			0.04265			
H3.3-Cas9	GRN-2	0.8515			20.17			0.04221			
H3-Cas9	GRN-g2	0.7219	0.8289	0.06183	20.72	18.77	1.478	0.03484	0.04484	0.005185	0.10652073
H3-Cas9	GRN-g2	0.9361			19.72			0.04746			
H3-Cas9	GRN-g2	0.8288			15.87			0.05222			
H3.1-Cas9	GRN-g2	0.6754	0.73	0.178	21.67	19.39	1.85	0.03116	0.037	0.007072	0.05875435
H3.1-Cas9	GRN-g2	1.062			20.79			0.05108			
H3.1-Cas9	GRN-g2	0.4526			15.73			0.02877			
H3.2-Cas9	GRN-g2	0.649	0.8324	0.1029	17.11	20.06	1.501	0.03793	0.04121	0.002298	0.03488088
H3.2-Cas9	GRN-g2	1.005			22.02			0.04564			
H3.2-Cas9	GRN-g2	0.8434			21.05			0.04006			
H3.3-Cas9	GRN-g2	0.5142	0.4785	0.09388	7.545	8.406	0.4305	0.06815	0.05749	0.01176	0.79188752
H3.3-Cas9	GRN-g2	0.3011			8.852			0.03401			
H3.3-Cas9	GRN-g2	0.6204			8.821			0.07033			
