# Supplementary material for: Fusion of histone variants to Cas9 suppresses non-homologous end joining
Source: PLoS One. 2024 May 13;19(5):e0288578. doi: 10.1371/journal.pone.0288578 (PMC11090291; doi:10.1371/journal.pone.0288578)
Supplement: S15 Table — (PDF) [file pone.0288578.s018.pdf]

S15 Table. Digital PCR raw data of Fig 5B.

Sample Name	gRNA	HDR frequency (%)	HDR average frequency (%)	HDR S.E. (%)	NHEJ frequency (%)	NHEJ average frequency (%)	NHEJ S.E. (%)	HDR / NHEJ	HDR / NHEJ average	HDR / NHEJ S.E.	Fold increase compared to N-GS3	Fold increase compared to Cas9
Cas9	ATP7B-3	0.7752	0.6989	0.07204	15.61	15.86	0.135	0.04964	0.04408	0.004612	0.3252	
Cas9	ATP7B-3	0.5549			15.88			0.03492				
Cas9	ATP7B-3	0.7666			16.08			0.04767				
H2A.X (N-GS3)	ATP7B-3	1.636	1.199	0.2201	15.16	14.96	0.1987	0.1079	0.08002	0.01418		1.815
H2A.X (N-GS3)	ATP7B-3	1.028			14.56			0.07059				
H2A.X (N-GS3)	ATP7B-3	0.9337			15.16			0.06157				
H2A.1-Cas9	ATP7B-3	0.9419	0.7457	0.1246	15.2	14.94	0.1855	0.06197	0.04989	0.008229	0.1399	1.132
H2A.1-Cas9	ATP7B-3	0.7808			14.58			0.05353				
H2A.1-Cas9	ATP7B-3	0.5145			15.05			0.03416				
Assay background	ATP7B-3	0.08394	0.05615	0.01463	0.1838	0.218	0.1034	0.4565				
Assay background	ATP7B-3	0.03431			0.05826			0.5888				
Assay background	ATP7B-3	0.05019			0.4118			0.1218				
Cas9	ATP7B-g3	3.745	4.871	0.7288	36.1	37.75	0.8368	0.1037	0.1284	0.01683	0.9365	
Cas9	ATP7B-g3	6.236			38.83			0.1605				
Cas9	ATP7B-g3	4.633			38.31			0.1209				
H2A.X (N-GS3)	ATP7B-g3	4.287	3.864	0.2162	27.93	28.19	0.2053	0.1534	0.1371	0.008468		1.068
H2A.X (N-GS3)	ATP7B-g3	3.728			28.04			0.1329				
H2A.X (N-GS3)	ATP7B-g3	3.576			28.59			0.125				
H2A.1-Cas9	ATP7B-g3	4.03	3.316	0.433	28.5	25.92	1.304	0.1414	0.127	0.01145	0.5186	0.9891
H2A.1-Cas9	ATP7B-g3	2.535			24.27			0.1044				
H2A.1-Cas9	ATP7B-g3	3.384			25			0.1353				
Assay background	ATP7B-g3	0.0478	0.03568	0.0941	0.08332	0.0358	0.02376	5.608				
Assay background	ATP7B-g3	0.04209			0.01246			3.376				
Assay background	ATP7B-g3	0.01715			0.01164			1.473				
Cas9	APOE-g1	10.53	10.89	0.1855	9.408	9.22	0.418	1.119	1.187	0.06841	0.8243	
Cas9	APOE-g1	11.15			8.42			1.324				
Cas9	APOE-g1	10.99			9.831			1.118				
H2A.X (N-GS3)	APOE-g1	8.157	9.382	0.6309	6.164	6.519	0.371	1.323	1.44	0.07726		1.213
H2A.X (N-GS3)	APOE-g1	9.731			6.133			1.586				
H2A.X (N-GS3)	APOE-g1	10.25			7.261			1.412				
H2A.1-Cas9	APOE-g1	8.543	8.854	0.1569	4.79	5.719	0.473	1.783	1.566	0.1111	0.4073	1.319
H2A.1-Cas9	APOE-g1	8.973			6.338			1.415				
H2A.1-Cas9	APOE-g1	9.045			6.03			1.499				
Assay background	APOE-g1	0.05614	0.04155	0.01569	0.5088	0.5524	0.07844	0.1103				
Assay background	APOE-g1	0.05833			0.7047			0.08277				
Assay background	APOE-g1	0.01019			0.4437			0.02298				
