# Supplementary material for: Fusion of histone variants to Cas9 suppresses non-homologous end joining
Source: PLoS One. 2024 May 13;19(5):e0288578. doi: 10.1371/journal.pone.0288578 (PMC11090291; doi:10.1371/journal.pone.0288578)
Supplement: S16 Table — (PDF) [file pone.0288578.s019.pdf]

S16 Table. Frequency of modified sequences detected at off-target loci.

gRNA	Name	Plasmid	modified (%)
RBM20-2	OTS1	Cas9	2.03
		H2A.1-Cas9	1.9
	OTS2	Cas9	2.85
		H2A.1-Cas9	2.75
	OTS3	Cas9	3.4
		H2A.1-Cas9	4.11
RBM20-g1	OTS4	Cas9	3.9
		H2A.1-Cas9	2.49
	OTS5	Cas9	3.15
		H2A.1-Cas9	2.52
	OTS6	Cas9	9.79
		H2A.1-Cas9	11.34
GRN-2	OTS7	Cas9	2.43
		H2A.1-Cas9	2.5
	OTS8	Cas9	2.42
		H2A.1-Cas9	2.69
	OTS9	Cas9	5.21
		H2A.1-Cas9	3.47
GRN-g2	OTS10	Cas9	3.97
		H2A.1-Cas9	2.15
	OTS11	Cas9	4.5
		H2A.1-Cas9	1.92
	OTS12	Cas9	3.71
		H2A.1-Cas9	2.01
ATP7B-3	OTS13	Cas9	2.84
		H2A.1-Cas9	2.39
	OTS14	Cas9	2.26
		H2A.1-Cas9	2.58
	OTS15	Cas9	4.43
		H2A.1-Cas9	4.51
ATP7B-g3	OTS16	Cas9	4.02
		H2A.1-Cas9	3.95
	OTS17	Cas9	2.38
		H2A.1-Cas9	2.24
	OTS18	Cas9	2.56
		H2A.1-Cas9	2.65
APOE-g1	OTS19	Cas9	3.07
		H2A.1-Cas9	3.13
	OTS20	Cas9	2.96
		H2A.1-Cas9	2.95
	OTS21	Cas9	3.06
		H2A.1-Cas9	3.43
